# Automated Imaging of Cataract Surgery Using Artificial Intelligence

**DOI:** 10.3390/diagnostics15040445

**Published:** 2025-02-12

**Authors:** Young Jae Kim, Sung Ha Hwang, Kwang Gi Kim, Dong Heun Nam

**Affiliations:** 1 Gachon Biomedical & Convergence Institute, Gil Medical Center, Gachon University, Incheon 21565, Republic of Korea; kimyj10528@gmail.com; 2 Department of Ophthalmology, Gil Medical Center, College of Medicine, Gachon University, Incheon 21565, Republic of Korea; sungha@gilhospital.com; 3 Department of Biomedical Engineering, Gil Medical Center, College of Medicine, Gachon University, Incheon 21565, Republic of Korea

**Keywords:** cataract surgery, parameter estimation, Pix2Pix, ensemble learning, ResNet-50, regression

## Abstract

**Objectives:** This study proposes a state-of-the-art technology to estimate a set of parameters to automatically display an optimized image on a screen during cataract surgery. **Methods:** We constructed an architecture comprising two stages to estimate the parameters for realizing the optimized image. The Pix2Pix approach was first introduced to generate fake images that mimic the optimal image. This part can be considered a preliminary step; it uses training datasets comprising both an original microscopy image as the input data and an optimally tuned image by ophthalmologists as the label data. The second part of the architecture was inspired by ensemble learning, in which two ResNet-50 models were trained in parallel using fake images obtained in the previous step and unprocessed images. Each set of features extracted by the ensemble-like scheme was exploited for the regression of the optimal parameters. **Results:** The fidelity of our method was confirmed through relevant quantitative assessments (NMSE 121.052 ± 181.227, PSNR 29.887 ± 4.682, SSIM 0.965 ± 0.047). **Conclusions:** Subsequently, surgeons reassured that the objects to be highlighted on the screen for cataract surgery were faithfully visualized by the automatically estimated parameters.

## 1. Introduction

Cataract surgery is one of the most common surgeries performed worldwide [1]. Owing to the help of several ophthalmologists and biomedical engineers, the technology and equipment associated with cataract surgery have remarkably improved, and 3D heads-up visualization systems are now being used [2]. This system has several advantages over traditional methods performed through a microscope and can provide a three-dimensional appearance of the crystalline lens and a far more enhanced depth of focus, which greatly improves visibility [3,4,5]. In addition, medical assistants and nurses can check the progress of the operation on the same screen as the operator, allowing smoother communication during the surgery. It can also be appreciated as an advanced piece of equipment in terms of ergonomics because the operator is able to watch the video with his head up comfortably [6,7].

In cataract surgery using a 3D heads-up visualization system, it is crucial to appropriately adjust the screen parameters so that the lens and surrounding structures can be clearly observed [8,9]. However, a uniformly imaged screen cannot guarantee optimal visibility, because the types and grades of cataracts vary from person to person. In addition, the process of obtaining an optimized image by manipulating various sets of parameters in real time is time-consuming and cumbersome. Considering the limitations and inconveniences stated previously, it would be of great help to introduce a system that automatically calculates and applies parameters optimized for image visibility according to the type and grade of each individual’s cataract.

Recently, deep learning-based medical image reconstruction has been extensively studied, and many studies have been dedicated to the field of ophthalmology [10,11,12,13,14]. Nevertheless, to the best of our knowledge, there has been a lack of research related to optimizing surgical images based on deep learning in 3D heads-up visualization systems for cataract surgery. This study proposes an approach to extract and apply image parameters optimized for individuals through raw images before commencing cataract surgery using a deep learning-based optimal image visualization prediction model.

Predicting the parameters consisting of continuous values is equivalent to solving a linear regression problem using features extracted from raw microscopic images. Accordingly, our ultimate goal to construct a model that faithfully maps a feature domain to a parameter domain, eliciting only significant features that effectively represent the characteristics of the raw datasets could be viewed as a key process for enhancing the accuracy of the regression model to be built. This objective was attained by regressing a predefined parameter set on the features extracted from two independently trained individual residual networks; each output yielded by the two networks was combined into a one-dimensional array and sent to a fully connected layer to estimate the optimal parameters. The parameters determined using the proposed regression framework reproduced a surgical image that was perceptually and numerically comparable to a reference image optimally tuned by surgeons. To acquire promising results, we leveraged the aforementioned ensemble-like model by concurrently analyzing two heterogeneous datasets, i.e., raw images and fake images produced by generative adversarial networks (GANs).

The remainder of this paper is organized as follows: In Section 2, coupled with an explanation of the comprehensive experimental procedure, the key concepts supporting the neural network devised in this study are detailed. Afterwards, Section 3 delivers a qualitative and quantitative assessment of the resulting images, followed by Section 4 that discusses the contribution, novelty, and limitations of our work. Finally, we conclude this paper with a summary and suggestions for future research.

## 2. Materials and Methods

### 2.1. Data Preparation

This study was approved by the Institutional Review Board (GDIRB2023-179) of Gachon University and was performed in accordance with the tenets of the Declaration of Helsinki. All the participants provided written informed consent.

**Figure 1:**
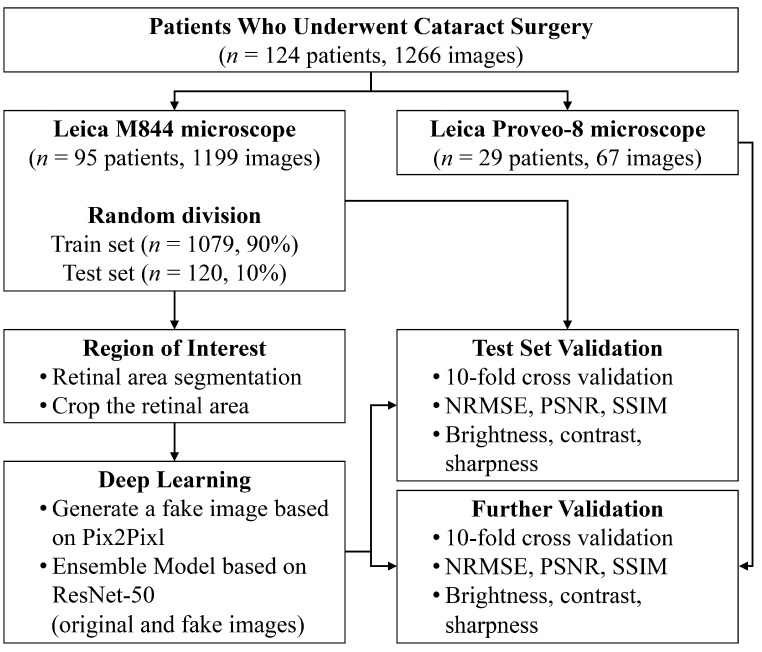
Flowchart of the parameter estimation process for optimized imaging in cataract surgery.

A total of 1199 ocular microscopic images from 95 patients were acquired during ocular surgery at Gachon University. Ocular microscopic images were acquired using the NGENUITY 3D visualization system (Alcon, Fort Worth, TX, USA) combined with a Leica M844 microscope (Leica Microsystems, Wetzlar, Germany). For further validation, 67 data points from 29 patients taken with different microscopes were collected at the Gachon University Gil Hospital. Data for further validation were acquired using a Leica Proveo-8 microscope (Leica Microsystems, Wetzlar, Germany), which is a newer model than the M844 (Figure 1).

**Figure 2:**
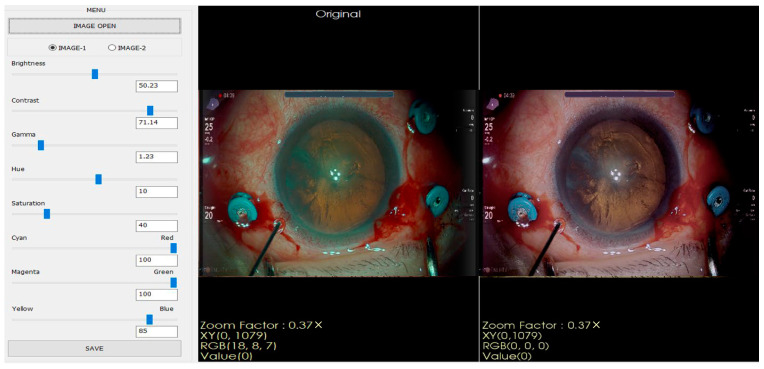
The leftmost panel contains scroll bars to adjust the eight parameters, and the two photos positioned in the middle and the right display an unprocessed image and a manually optimized image, respectively.

From these images, ophthalmologists recorded eight control parameters: brightness, saturation, contrast, cyan, gamma, magenta, hue, and yellow, enabling the realization of an optimal visual configuration for highlighting the retinal areas by manually adjusting the microscope, as shown in Figure 2.

The manual tuning procedures for acquiring optimal surgical images were performed by a physician (D.H.N.) using the NGENUITY 3D visualization and operating microscope. The surgical technique described in our previous studies was used in all cataract surgeries with an illuminated chopper (Nam illumination probe with chopper, Oculight, Republic of Korea) [15,16,17].

The 1199 sets of parameters collected served as the reference for estimating the optimal parameter set. To address overfitting during the training process, 1199 pairs of images and parameter sets were split into a validation set and a training set with a ratio of 1 to 9 equivalent to 10-fold cross-validation.

### 2.2. Segmentation of Region of Interest

First, to enhance the estimation accuracy of the parameters, we segmented only the retinal area of interest from an entire image by excluding unwanted information such as the sclera, trocars, and user interface (UI) texts. For the implementation of the process, we took advantage of relevant built-in functions provided in OpenCV and scikit-image libraries. The protocol for obtaining the region of our interest (ROI) is described as follows.

Color images were first converted to grayscale images, using which we determined a threshold value to distinguish the distribution of the retinal area from the background by means of histogram analysis [18]. Using the threshold, the grayscale images were again transformed to binary images expressed in white, including the ROI, and black, representing the areas to be removed. Subsequently, the adjacent white pixels merge with each other, and consequently, several chunks of white shapes appear in the entire image. Among the multiple white objects, the greatest one was considered the retinal area, and other white pieces were turned into black pixels, where identifying the size of objects and screening the most probable retinal area were performed using the remove small objects method provided in the morphology computation package of scikit-image open-source libraries [19]. Finally, the right, left, top, and bottom coordinates of the white single object representing the ROI were used to crop the retinal area in the color images. The cropped images were uniformly resized to a resolution of 512 × 512 pixels, and the pixel intensity was set in the range of 0 to 255. However, because the values of each parameter were scaled in different ranges, we normalized the ranges of individual types of parameters from zero to one [20].

### 2.3. Proposed Framework

Various deep learning architectures that have evolved based on a convolutional neural network have demonstrated the possibility of their application as a reliable solver in diverse fields, such as image classification, semantic image segmentation, and medical image analysis, and their success is known as being attributed to their efficient and powerful feature extraction ability [21,22,23]. We adopted ResNet-50 for feature extraction from input images. ResNet, which is a short residual neural network, is able to learn more complex features because it enables models to train in far deeper layers by introducing residual blocks that mitigate gradient vanishing [24].

On the other hand, to form a more robust feature domain, we trained the identical ResNet-50 model in parallel to the one stated above, which uses fake images produced by the Pix2Pix approach as input datasets. This approach was developed to address the image-to-image translation problem as a member of the GAN family. Our study exploited the model to replicate manually tuned optimal images [25,26]. Table 1 provides the layers and parameters for the Pix2Pix model. We expected that concurrently using the features acquired from the fake images would lead to a more desirable parameter estimation than using features only taken from the raw microscopic images because the fake images are capable of successfully mimicking images adjusted by the optimal parameters.

The features extracted from the two ResNet-50 models independently trained were integrated into a single vector, which was subsequently fed into the fully connected layer via linear regression. The design of the proposed architecture can be viewed as an ensemble technique in the sense that the output from two independent models simultaneously contributes to the estimation of the parameters.

**Figure 3:**
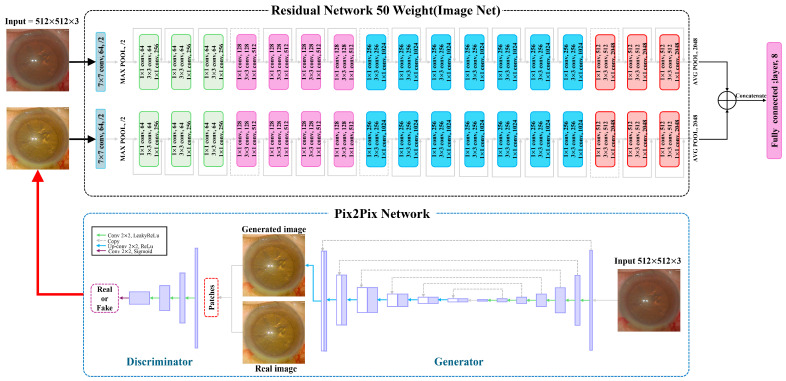
The overall framework devised in this work is described. The upper network represents the ensemble model for the prediction of parameters, and the lower shows a schematic diagram of the production of fake images by the Pix2Pix approach.

**Figure 4:**
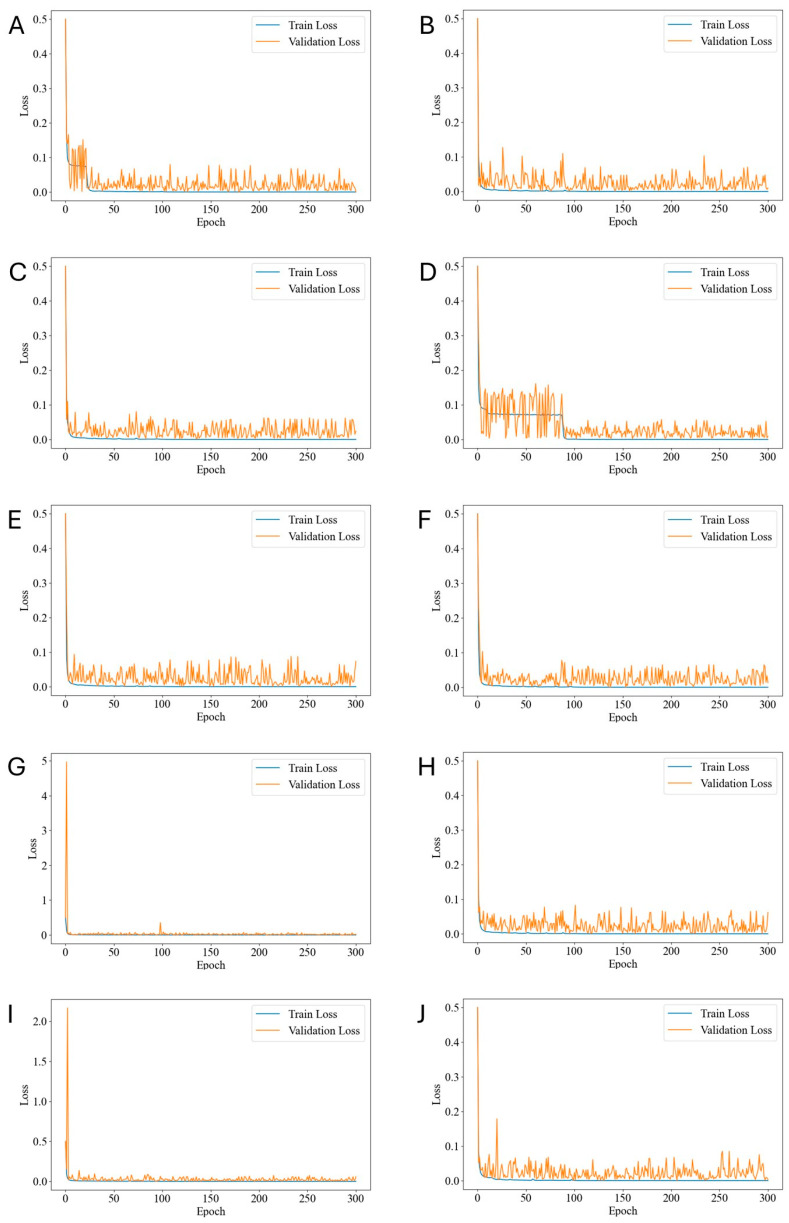
Graph showing the changes in the loss function during the training process for each cross-validation fold. (**A**) Fold 1, (**B**) Fold 2, (**C**) Fold 3, (**D**) Fold 4, (**E**) Fold 5, (**F**) Fold 6, (**G**) Fold 7, (**H**) Fold 8, (**I**) Fold 9, (**J**) Fold 10.

The overall workflow is depicted in Figure 3, and the changes in the loss function during the training process for each cross-validation fold are shown in Figure 4.

### 2.4. Metrics

In this study, the performance of the proposed algorithm was evaluated using normalized root mean square error (NRMSE), peak signal-to-noise ratio (PSNR), and structural similarity index measure (SSIM). The NRMSE measures the difference between the raw and restored images, with values close to zero indicating little or no difference. PSNR measures the signal-to-noise ratio between the raw and restored images, with higher values indicating a smaller difference. The SSIM measures the structural similarity between raw and restored images, with values close to 1 indicating little to no difference [27].

Furthermore, for quantitative analysis of visualization, brightness, contrast, and sharpness were measured and compared. Brightness represents the overall brightness level of an image, with higher values indicating brighter images and lower values indicating darker images [28]. The contrast represents the difference in brightness between adjacent pixels in an image, with higher values indicating greater contrast and lower values indicating less distinct differences in brightness [29]. Sharpness indicates the clarity and crispness of an image; higher values indicate sharper edges and boundaries [30].

The following equations show the calculation process for each metric: *N* represents the total number of pixels. *I* represents the reference image obtained by applying the eight parameters manually adjusted by an ophthalmologist, and *P* represents the resulting image obtained by applying the eight predicted parameters to the original image using the proposed method. *I_max_* and *I_min_* represent the brightest and darkest values in image *I*, respectively. $L\left(I,P\right)$ represents the brightness similarity between *I* and *P*, *C*$\left(I,P\right)$ represents the contrast similarity between *I* and *P*, and $S\left(I,P\right)$ represents the structural similarity between *I* and *P*. α is the weighting parameter. μI and μP are the means of the brightness of images *I* and *P*, respectively. σI and σP are the standard deviations of the contrast of images *I* and *P*, respectively. σIP is the covariance of images *I* and *P*. $C_{1}$, $C_{2}$, and $C_{3}$ represent constants.

(1) $$NRMSE=\frac{\sqrt{\frac{\sum {\left(I-P\right)}^{2}}{N}}}{I_{max}+I_{min}}$$

(2) $$PSNR=\frac{{I_{max}}^{2}}{\frac{1}{N}\sum {\left(I-P\right)}^{2}}$$

(3) $$SSIM={\left[L\left(I,P\right)\times C\left(I,P\right)\times S\left(I,P\right)\right]}^{\alpha }$$

(4) $$L\left(I,P\right)=\frac{2\mu I\mu P+C_{1}}{\mu I^{2}\mu P^{2}+C_{1}}$$

(5) $$C\left(I,P\right)=\frac{2\sigma I\sigma P+C_{2}}{\sigma I^{2}\sigma P^{2}+C_{2}}$$

(6) $$S\left(I,P\right)=\frac{2\sigma IP+C_{3}}{\sigma I\sigma P+C_{3}}$$

## 3. Results

**Figure 5:**
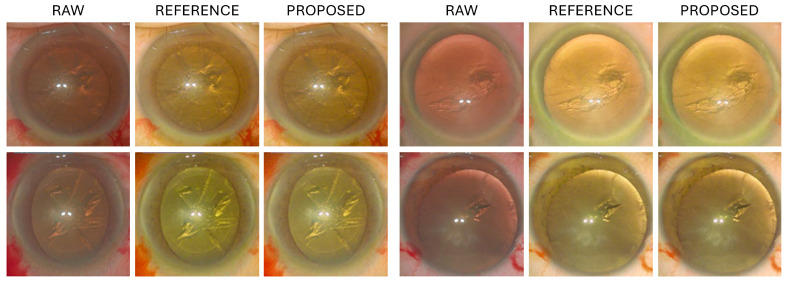
Results of applying the 8 parameters predicted by the proposed method to a test set (M844 microscope).

**Figure 6:**
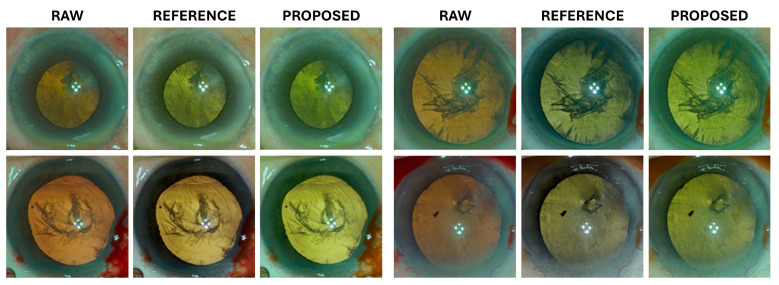
Results of applying the 8 parameters predicted by the proposed method to a test set (Proveo-8 microscope) for further validation.

In this study, we utilized eight parameters that were manually adjusted by an ophthalmologist (reference images) and eight parameters predicted using our proposed method. These parameters were applied to raw images of a distinct test set. Subsequently, a comparative analysis of the resulting images was performed. The images generated by the proposed method exhibited results that were visually similar to the reference images across both the M844 and Proveo-8 datasets. Figure 5 and Figure 6 show the resulting images on the M844 and Proveo-8 datasets, respectively.

To evaluate the performance of the proposed model, we measured NRMSE, PSNR, and SSIM using a separately constructed test set (Table 2). The results demonstrate that the M844 dataset achieved good performance across all three metrics, with NRMSE, PSNR, and SSIM values of 0.09 ± 0.06, 29.89 ± 4.68, and 0.97 ± 0.05, respectively. Conversely, the Proveo-8 dataset exhibited lower performance compared to the M844 data, with NRMSE, PSNR, and SSIM values of 0.23 ± 0.09, 22.63 ± 2.99, and 0.92 ± 0.05, respectively.

For a quantitative analysis of the visualization, we measured the brightness, contrast, and sharpness and compared them to the raw and reference images (Table 3). The results indicate that, in the M844 dataset, the reference image shows significantly higher brightness, contrast, and sharpness values than the raw image (*p* < 0.001). Furthermore, the proposed model exhibited significantly higher values for all metrics compared with the raw image (*p* < 0.001). When compared with the reference image, the proposed model performed similarly, with no statistically significant differences in brightness (*p* = 0.306), contrast (*p* = 0.368), or sharpness (*p* = 0.203). In the case of the Proveo-8 dataset, the reference image demonstrated significantly higher values than the raw image in terms of contrast and sharpness (*p* < 0.001), but not brightness (*p* = 0.097). Additionally, the proposed model displayed significantly higher values for all metrics compared to the raw image (*p* < 0.001). Notably, in contrast to the M844 dataset, the proposed model exhibited significantly higher or lower values (*p* < 0.001) in terms of brightness, contrast, and sharpness compared with the reference image.

## 4. Discussion

Cataract surgery is performed in several stages, each of which requires a dedicated, optimized image. More specifically, for continuous curvilinear capsulorhexis, the region of the anterior capsule should be displayed with an obvious contrast to other objects. For phacoemulsification, it is necessary to highlight the thickness and structure of the lens. However, when performing lens capsule polishing, the anterior and posterior capsules should be clearly visualized [31,32,33]. We successfully revealed that tedious parameter-tuning processes relying only on manual work could be automated while ensuring a quality equivalent to the level of hand operation. Moreover, the most promising benefit of this study is that the optimization process is instantaneously realized.

One of the main limitations of our model is that it was exclusively trained on data captured using the M844 equipment. This dependency on training data poses a potential weakness. To address this limitation, we conducted an evaluation using images from a different equipment, namely, the Proveo-8. This dataset was not utilized during the training process and served as a means of assessing the generalization capabilities of our model. The results indicate that our model achieved a high level of similarity with the reference images for both the M844 (SSIM: 0.97 ± 0.05) and Proveo-8 data (SSIM: 0.92 ± 0.05). In the quantitative analysis of visualizations aimed at assessing the visibility improvement, our model exhibited higher brightness, contrast, and sharpness values than the raw images in both the M844 and Proveo-8 datasets. However, the high values alone do not guarantee optimal visibility. The objective of our model is to closely approximate a manually manipulated image of optimal quality. Therefore, the closer the brightness, contrast, and sharpness values align with those of the reference image, the closer we achieve optimal quality.

For the M844 dataset, our model demonstrated minimal differences in brightness (*p* = 0.306), contrast (*p* = 0.368), and sharpness (*p* = 0.203) compared with the reference image. Conversely, for the Proveo-8 dataset, significant differences were observed in brightness (*p* < 0.001), contrast (*p* < 0.001), and sharpness (*p* < 0.001) compared to the reference image. These findings suggest that the proposed model successfully replaces the traditional manual manipulation of the M844 data, indicating optimal quality. However, the results did not meet the expectations of the Proveo-8 data in terms of achieving optimal-quality images. Nevertheless, the relatively good SSIM scores indicate the potential of the proposed model for new equipment and third-party equipment data. The limitations can be addressed in the future by collecting training data from various types of equipment.

These limitations can be addressed by collecting training data from a broader range of equipment in the future and further training the model. Data obtained from various types of devices and diverse patient populations can foster more generalized learning. Accordingly, we plan to acquire and train additional data from multiple institutions using different equipment and subsequently validate the performance of our model.

Our model has the advantage of being deployable in real surgical environments at any time, provided the equipment used matches that employed during training. Although it can be applied to devices not included in the training dataset—such as the Proveo-8 microscope used in this study—it is challenging to guarantee performance levels that meet expectations. Therefore, to deploy the model in an environment other than the M844 microscope, additional training must be conducted using images captured from the new equipment.

Furthermore, deploying the model on the surgical equipment itself (i.e., on-device) typically requires collaboration with the manufacturer, which may pose limitations due to conflicts of interest or other constraints. To circumvent these issues, one can instead use a dedicated computer or laptop. By connecting an external computer to the microscope to receive real-time images, the model can predict and provide optimal parameters. This approach allows indirect use of our model without direct collaboration from the equipment manufacturer.

## 5. Conclusions

We propose a deep learning-based model for predicting parameters, aiming to provide optimized images for cataract surgery. The results demonstrated a high level of performance and visualization quality. If the limitations concerning model generalization can be addressed through further research, it holds the potential for application in cataract surgery, offering valuable assistance to numerous ophthalmic surgeons.

## Figures and Tables

**Table 1:** Summary of the Pix2Pix model architecture.

Layer	Type	Output Shape	Activation	Parameters
0	Input Layer	(512, 512, 3)	-	0
1	Conv2D	(256, 256, 64)	LeakyReLU	3136
2	Conv2D	(128, 128, 128)	LeakyReLU	131,200
3	Conv2D	(64, 64, 256)	LeakyReLU	524,544
4	Conv2D	(32, 32, 512)	LeakyReLU	2,097,664
5	Conv2D	(16, 16, 512)	LeakyReLU	4,194,816
6	Conv2D	(8, 8, 512)	LeakyReLU	4,194,816
7	Conv2D	(4, 4, 512)	LeakyReLU	4,194,816
8	UpSampling2D	(8, 8, 512)	-	0
9	Conv2D	(8, 8, 512)	ReLU	4,194,816
10	UpSampling2D	(16, 16, 512)	-	0
11	Conv2D	(16, 16, 512)	ReLU	8,389,120
12	UpSampling2D	(32, 32, 512)	-	0
13	Conv2D	(32, 32, 512)	ReLU	8,389,120
14	UpSampling2D	(64, 64, 512)	-	0
15	Conv2D	(64, 64, 256)	ReLU	4,194,560
16	UpSampling2D	(128, 128, 256)	-	0
17	Conv2D	(128, 128, 128)	ReLU	1,048,704
18	UpSampling2D	(256, 256, 128)	-	0
19	Conv2D	(256, 256, 64)	ReLU	262,208
20	UpSampling2D	(512, 512, 64)	-	0
21	Conv2D	(512, 512, 3)	Tanh	6147

**Table 2:** Quantitative performance evaluation of the proposed model on a test set.

	NRMSE	PSNR	SSIM
M844	0.09 ± 0.06	29.89 ± 4.68	0.97 ± 0.05
Proveo-8	0.23 ± 0.09	22.63 ± 2.99	0.92 ± 0.05

**Table 3:** Quantitative analysis of three visualization metrics: brightness, contrast, and sharpness.

	Raw	Reference	Proposed	*p* *	*p* ^#^	*p* ^†^
Brightness						
M844	28.02 ± 9.51	37.70 ± 13.23	38.28 ± 14.22	<0.001	<0.001	0.306
Proveo-8	14.30 ± 3.71	13.95 ± 4.14	17.52 ± 4.66	0.097	<0.001	<0.001
Contrast						
M844	37.03 ± 6.48	45.86 ± 8.39	46.16 ± 8.02	<0.001	<0.001	0.368
Proveo-8	28.66 ± 4.37	30.20 ± 5.45	33.90 ± 5.58	<0.001	<0.001	<0.001
Sharpness						
M844	0.94 ± 0.06	4.65 ± 1.39	4.72 ± 1.14	<0.001	<0.001	0.203
Proveo-8	3.64 ± 0.87	5.25 ± 1.18	4.91 ± 1.13	<0.001	<0.001	<0.001

* *t*-test between the raw and reference images. ^#^ *t*-test between the raw image and the proposed method. ^†^ *t*-test between the reference image and the proposed method.

## Data Availability

The data presented in this study are available on request from the corresponding author due to ethical reasons.
